# Epidemiology of Accidents and Traumas in Qom Province in 2010

**DOI:** 10.5812/atr.8382

**Published:** 2013-12-01

**Authors:** Moharram Karami Joushin, Abedin Saghafipour, Mehdi Noroozi, Hamid Soori, Esmaeil Khedmati Morasae

**Affiliations:** 1 Health Deputy, Qom University of Medical Sciences, Qom, IR Iran; 2 Department of Epidemiology, Shahid Beheshti University of Medical Sciences, Tehran, IR Iran; 3 Department of Health Education, Tehran University of Medical Sciences, Tehran, IR Iran; 4 Center for Community Based Participatory Research, Tehran University of Medical Sciences, Tehran, IR Iran

**Keywords:** Trauma, Epidemiology, Injury, Qom

## Abstract

**Background:**

Accidents are the most important public health challenges in our society. To prevent the accidents, the identification of their epidemiological features seems necessary.

**Objectives:**

This study was conducted to reveal the epidemiological features of accidents and their casualties in Qom province in 2010.

**Patients and Methods:**

A cross–sectional study was conducted on 29426 injured people referred to Qom province hospitals in 2010. Information about place, time, type of accidents and traumas and demographic variables had been collected in a veteran hospital. Data were analyzed by SPSS (version 16) software, using chi-square test and logistic regression.

**Results:**

The incidence of accidents was about 27/1000 per year. The incidences of traffic accidents, motorcycle accidents, violence, burns, poisoning and suicides were 3, 1.6, 1.2, 0.3, 0.8, 0.37 cases per 1000 people respectively. Strikes (65%) and falls (12%) were the main causes of traumas. Forty-six percent of all injuries had occurred in 16 - 30 years groups. Most frequent accidents were as follows: fall (97%) and strike (50%) in < 12, violence (46%) in 20 - 29, suicide (71%) in 15 - 29, poisoning (34%) and burns (20%) among < 5 years old. Pedestrian and motorcycle accidents among +60 years old people were significantly higher than other (P = 0.000). Odds ratio for suicide among female was about 3.36 and in 16 - 30 age-group was 15.7 more than +60 years old group (P = 0.000).

**Conclusions:**

Most traumas in Qom province occurred among younger age-groups and strikes and falls are the main causes of such traumas. Therefore, safeties to prevent falls and traffic regulations to reduce strikes can be effective strategies.

## 1. Background

According to global statistics, 5.7 million people lost their lives due to injuries in 2004, which was about 9.8% of all death causes (1). It is estimated that 16000 persons die from injuries every day. Injuries are the most common causes of death among 15 - 59 year old people, and men have higher death rates than women. According to a study in Iran, 28% of the total number of disability adjusted life years is caused by injuries so that the highest rate of mortality among people of 5 - 44 years old is related to number of injuries (2). Home is the main site of injuries in most of the cases. Approximately a fifth of all unintentional injuries occur at home (3). Several studies in Iran have shown that the home accidents are the second major cause of injuries, after traffic accidents (4, 5). The majority of people experienced traumas in Kashan have primary or secondary school education and most of them are housewives. Also, falling is the most common cause of injuries among young men and older women (6). In a study on home accidents, Khosravi has shown that three leading causes of injury are burns, injuries (due to strike of sharp objects) and falls respectively (7). Another study on home-related injuries in Iran showed that children aged 0 – 4 years old have the highest rate of injury and the elderly (≥ 60 years) have the lowest rates. This matter varied between the sexes; most patients under 15 years old were male, but female showed the highest rate in different age groups. Accordingly, burns, falls, and poisoning were the three most common causes of death (8).

Accidents are a very important cause of death and disability worldwide so that WHO has decided to select this topic as a major area of further research and discussion. It is expected that injury-related burden of disease and mortality will have been increased by 2020. In particular, burden of road traffic accidents, self-violence, violence against others, and war will have been increased substantially (9). In a study in Rafsanjan (center of Iran), it was revealed that road accidents and falls were the main causes of accidents (10). In a similar study in Kashan, it was found that about 50% of accidents were traffic accidents. Also, it was revealed that men who were 10 - 30 years old and women under 50 years old were more prone to accidents (11). The number of people residing in Qom province in 2010 was about one million, of which 94% resided in urban areas. Nevertheless, there has been no study yet to precisely look over accident-related issues in the province that lies at the center of national traffic network.

## 2. Objectives

This study, for the first time, aimed to assess the epidemiologic features of accidents and traumas in Qom. We believe that our study can be a help-guide to determine the best place, time and age-group to direct interventional programs . It can improve the effectiveness of interventional strategies.

## 3. Patients and Methods

The study was a descriptive - analytical one in which the recorded data of 29426 injured people admitted to hospitals in Qom province in 2010 were analyzed. The related data have been collected from a system to collect the data of accident victims in veteran hospitals. This national registration system designed by Center of Disease Control of the Ministry of Health is in Access program format containing the variables of age, sex, region, event, event location, event type, date of event, outcome of the event, and name of recording center. This system now records the information of all patients admitted to the emergency wards of all hospitals around the country. In the present study, confidentiality of patient' information was considered and no name was mentioned in used questionnaires. Descriptive tests such as frequency tables and analytical tests such as Chi-square and logistic regression were used to analyze the data. (The level of P-value < 0.05 was considered as statistically significant).

## 4. Results

Totally, 29426 accident cases were recorded. Seventy percent of cases were male and accidents were mostly among single (71.4%), urban residents (94%) and people with lower levels of education (67%). Most of accidents took place in streets (65%) and homes (20%). Workplace (8%), public places (2%), schools and academic places (1%), recreational and sporting places (1%) and other places (3%) were placed in the following ranks. Mean (SD) age of the victims among men and women were 27 (17) and 31 (22) years respectively, which showed a significant difference (Table 1). 

**Table 1.** Distribution of Accidents According to the Age Groups and Gender

Accident	Age, Mean (SD), y	Age Groups							
		< 12 Child		13-19 Teenage		20-60 Adult		> 60 Old	
		Female, No. (%)	Male, No. (%)	Female, No. (%)	Male, No. (%)	Female, No. (%)	Male, No. (%)	Female, No. (%)	Male, No. (%)
**Fall**	< 1 (00.0)	1256 (34.6)	2282 (62.8)	23 (00.7)	24 (00.7)	7 (00.2)	12 (00.3)	15 (00.5)	5 (00.1)
**Strike**	11.8 (10.7)	3045 (16.0)	6470 (34.0)	663 (04.0)	2519 (13.0)	1471 (07.0)	5053 (26.0)	0 (00.0)	0 (00.0)
**Suicide**	30 (00.0)	0 (00.0)	0 (00.0)	0 (00.0)	0 (00.0)	117 (29.0)	290 (71.0)	0 (00.0)	0 (00.0)
**Violence**	32.4 (01.3)	0 (00.0)	0 (00.0)	0 (00.0)	0 (00.0)	346 (26.0)	961 (74.0)	0 (00.0)	0 (00.0)
**Pedestrian accidents**	35.4 (00.5)	3 (00.6)	6 (01.2)	5 (01.0)	7 (01.4)	126 (25.9)	299 (61.5)	3 (00.6)	2 (00.4)
**Motorcycle accident**	40.3 (02.5)	5 (00.3)	4 (00.2)	14 (00.8)	17 (01.0)	544 (31.5)	1137 (65.8)	2 (00.1)	5 (00.3)
**Car accident**	48.8 (02.25)	2 (00.2)	4 (00.4)	2 (00.2)	13 (01.2)	423 (40.1)	601 (57.0)	6 (00.6)	4 (00.4)
**Poisoning**	57.6 (03.25)	0 (00.0)	0 (00.0)	5 (00.6)	10 (01.1)	292 (33.2)	406 (46.1)	77 (08.8)	90 (10.2)
**Scorpion and snake bite**	64.7 (00.5)	0 (00.0)	1 (04.2)	0 (00.0)	1 (04.2)	2 (08.3)	3 (12.5)	7 (29.2)	10 (41.7)
**Electric shock**	65.4 (00.5)	1 (01.6)	1 (01.6)	0 (00.0)	4 (06.2)	3 (04.7)	6 (09.4)	24 (37.5)	25 (39.0)
**Burns **	69.9 (02.1)	5 (01.7)	11 (03.9)	1 (00.3)	3 (01.0)	7 (02.5)	13 (04.6)	113 (39.1)	131 (46.1)
**Another accidents**	76	13 (03.0)	9 (02.0)	0 (00.0)	0 (00.0)	0 (00.0)	0 (00.0)	181 (48.0)	178 (47.0)

In terms of the type of accidents and their frequency, the following trend was observed: Motorcycle accidents (6%), car accidents (4%), violence (4%), pedestrian accidents (2%), poisoning (3%), burns (1%), suicide (1%), and other accidents (1%). Taking the type of accidents into account, the most frequent accident types in each age-group were as follows: Most cases of falls (63%) and strikes (34%) occurred among under 12 year old male children. Traffic (car, motorcycle and pedestrian) related accidents, suicide, and violence has happened among 20 - 60 year old people. Generally, the frequency of these latter accidents among men was as two times higher as the frequency among women. Burns, scorpion stings and electric shocks mostly occurred among people over 60 years old.

In overall, the incidence of accidents was about 27 cases per thousand people each year. The incidences of traffic accidents, motorcycle accidents, violence, burns, poisoning and suicides were 3, 1.6, 1.2, 0.3, 0.8, and 0.37 per 1000 cases respectively.

Results of logistic regression, to assess the association between sex and age with the type of accidents (female older than 60 years old were known as the reference) are shown in Table 2. As it shows, being younger than 45 years old is significantly correlated to suicide and violence. Odds ratio of suicide in age group of 16 - 30 years old was 15.7 times, and in age group of 31 - 45 years old was 7.2 times higher compared to the age group of +60 years old (P = 0.000). Frequency of car accidents, traumas, and burns among people younger than 60 years old was significantly higher than people of +60 years old, whereas frequency of pedestrian and motorcycle accidents ****among people ****under 60 years was significantly lower than group of + 60 years old (P = 0.000). Frequency of violence in the age group of 16 - 30 years old was 3.7 times, and in the age group of 31 - 45 years old was 3.1 times higher than ****people ****over 60 years old. Frequency of poisoning was significant only among people of 16 - 30 years old that was 2.3 times higher than in it among people of over 60 years old ****(P = 0.000). Frequency of burns among people ****under 15 years old was significantly remarkable (odds ratio). Frequency of injuries resulting from violence, electrocution, and motorcycle accidents were significantly higher among men. In contrast, suicide, falls, burns, car accidents and pedestrian accidents were significantly higher among women (P = 0.000). 

**Table 2.** The Logistic Regression for Assessing Association Between Sex and Age Groups with Type of Accidents

Sex and Age Groups	Suicide		Car Accident		Motorcycle Accident		Pedestrian Accidents		Poisoning		Violence		Burns		Fall		Strike	
	OR (P)	CI	OR (P)	CI	OR (P)	CI	OR (P)	CI	OR (P)	CI	OR (P)	CI	OR (P)	CI	OR (P)	CI	OR (P)	CI
**< 15**	3.14 (0.03)	1.10 - 8.80	1.34 (0.00)	1.13 - 1.60	0.23 (0.00)	0.21 - 0.24	0.65 (0.00)	0.55 - 0.75	0.90 (0.00)	0.40 - 2.00	0.52 (0.00)	0.34 - 0.80	3.70 (0.00)	2.70 - 5.00	0.72 (0.00)	0.65 - 0.80	3.00 (0.00)	2.80 - 3.30
**16 - 30**	15.7 (0.00)	5.80 - 42.00	1.74 (0.00)	1.48 - 2.05	0.40 (0.00)	0.37 - 0.43	0.55 (0.00)	0.48 - 0.64	2.30 (0.00)	1.40 - 5.60	3.70 (0.00)	2.60 - 5.30	1.70 (0.00)	1.30 - 2.40	0.35 (0.00)	0.31 - 0.39	2.40 (0.00)	2.30 - 2.60
**31 - 45**	7.20 (0.00)	2.60 - 20.00	1.79 (0.00)	1.51 - 2.12	0.36 (0.00)	0.33 - 0.38	0.58 (0.00)	0.50 - 0.68	1.40 (0.00)	0.70 - 3.20	3.10 (0.00)	2.20 - 4.50	2.00 (0.00)	1.50 - 2.70	0.45 (0.00)	0.40 - 0.50	2.50 (0.00)	2.30 - 2.70
**46 - 60**	1.22 (0.75)	0.36 - 4.10	1.44 (0.00)	1.19 - 1.74	0.50 (0.00)	0.46 - 0.54	0.79 (0.00)	0.67 - 0.93	1.40 (0.00)	0.70 - 3.20	1.70 (0.01)	1.10 - 2.60	1.90 (0.00)	1.30 - 2.60	0.58 (0.00)	0.51 - 0.66	2.00 (0.00)	1.80 - 2.20
**> 60** ^**a**^	1	-	1	-	1	-	1	-	1	-	1	-	1	-	1	-	1	-
**Male** ^**a**^	1	-	1	-	1	-	1	-	1	-	1	-	1	-	1	-	1	-
**Female**	3.36 (0.00)	2.70 - 4.10	1.31 (0.00)	1.22 - 1.40	0.70 (0.00)	0.68 - 0.72	1.40 (0.00)	1.30 - 1.50	2.00 (0.00)	1.50 - 2.60	0.39 (0.00)	0.33 - 0.46	1.20 (0.00)	1.10 - 1.30	1.19 (0.00)	1.12 - 1.27	1.07 (0.00)	1.03 - 1.10

^a^ reference groups.

According to regression analysis, pedestrian accidents were unevenly distributed throughout the year. A significant number of pedestrian accidents occur in autumn and summer. Interestingly, spring observed the lowest level of pedestrian accidents. Moreover, the number of motorcycle accidents in autumn was two times higher than the number in winter and spring. Violence in spring and winter was significantly lower than other seasons. The number of suicide cases had the highest frequency in summer. In terms of death cases per accident, 2% of poisoning, 0.02% of violence and 0.01% of car accidents led to loss of life.

## 5. Discussion

In the present study the incidence rate of injuries was about 27/1000 per year. In a study in Mazandaran (north of Iran) province in 2010, this rate was estimated to be about 23/1000 per year (12). In a similar study, Hosseini reported that urban accidents rate in Iran was about 126/1000 per year (13). The incidence rate of injury in adults from Kashan trauma survey was about 12.4/1000 per year (14).

In our study, 45% of all accidents had occurred among children under 12 years old. Almost all of incidents among children were falls and strikes. These findings are in accord with the results of researches conducted in Rafsanjan (10) and Kashan (11). In our study, Fazel et al. also showed that falls were the most common injury mechanism in home accidents (5). Of the reasons of higher frequency accidents among children, lack of adaptation to environmental risks can be numerated. Based on this study, 70 percent of the victims were male. This issue is similar to the findings of other studies (10-12). Among the possible reasons for this, more presence of men in activities of daily life can be emphasized.

Accidents occurred more among those with low education (66%), single (71%) and urban residents (95%). In the study of Bakhshi and colleagues, the victims of accidents in urban areas were almost as twice as the rural residents (10). Such a difference in frequency of accidents based on place of residence can be probably due to greater number of urban population in Qom province (94% of the population lives in the city).

In the present study, traumatic accidents were the first reason of events (65%). They were responsible for 32% of accidents in Mazandaran (12). In a study in Mazandaran province on the patterns of crash injuries among children under 15 years old, Ismaili et al. reported that falls (30.1%) and accidents (26.6%) were the most common type of incidents (15). The reason of this high frequency of traumatic events in the country, including Qom, is unknown and needs more research to determine the involved reasons. Traffic accidents accounted for 11% of all accidents in Qom city. In order to compare, various rates are reported in other studies; 50% in the Mazandaran (12), 41% in Chaharmahal Bakhtiari (west of Iran), 46% in Tehran (capital of Iran), 36% in Rafsanjan (south of Iran) (10) and 20% in Saudi Arabia (16).

In the present study, most traffic accidents were respectively motorcycle accidents (53%), car accidents (33%), and pedestrian accidents (14%). In a study by Taghipoor in Yazd province (center of Iran), pedestrian accidents were the most common traffic accidents with an incidence of 39% (17). Comparing to the rate of traffic accidents in our study, Moosazadeh and colleagues reported that the incidence of traffic accidents were about 9.2/1000 per year (12). It is worth to note that Iran has the first rank in number of incidents and related mortalities in the world.

The incidence of suicide in our study was about 0.37/1000 per year. This rate was about 1.06/1000 and 0.8/1000 in Mazandaran (12) and Qazvin (north of Iran) respectively. In our study, 58% of suicide cases significantly happened among the age group of 16 - 30 years old and women committed more suicidal attempts than men. This finding is consistent with other studies (10, 12). It seems that women are more fragile in stressful situations especially emotional stress. In the present study, the rate of injuries due to violence was about 1.2/1000 per year. Also, half of injury-related violence occurred among men of 16 - 30 years old. It is supposed that people in this age group has so much energy that should be consumed through positive social activities such as exercise, working, and by increasing the social welfare and so on.This rate in other studies was as follows; Bakhshi 6.9% (10), Moosazadeh 5.6% (12), and Karimi 5.1%. In our study as Qom is a religious city, rate of violence and suicide in it is remarkably lower than the rate in other cities.

In the present study, burns had an incidence rate of about 0.3/1000. This rate was much lower than that of Mazandaran (12) and Rafsanjan (10). Number of burns in autumn was lower than other seasons. We found that poisoning was the main cause of death due to accidents in Qom province. Death due to violence, falls, and traffic accidents came respectively after the poisoning. In a similar study traffic accident, strikes, suicide, and poisoning were the main causes of death respectively (12). This difference in main reasons of death can be due to deficit in precise record of accidents.

Taking the time of accidents, our study revealed that most of motorcycle accidents occurred in autumn and summer. This matter was in line with a study in Isfahan. In contrast, Falahzadeh has reported that most motorcycle accidents in Yazd occurred during the winter. A study in Kermanshah province (18) reported that most of motorcycle accidents had occurred in spring. In our study, suicide also was mostly committed in summer, which is in line with Naghavi's findings (18). In contrast, Moosazadeh reported that suicide was mostly committed in winter and spring in Mazandaran (12). In Qom province, most of traumas occur among younger age groups and strikes and falls are the main causes of traumas. Therefore, safety educations to prevent falls and traffic regulations to reduce strikes can be effective strategies.
